# Factors Associated With Open Access Publishing Costs in Oncology Journals: Cross-sectional Observational Study

**DOI:** 10.2196/44633

**Published:** 2023-03-16

**Authors:** Alex Koong, Ulysses Grant Gardner, Jason Burton, Caleb Stewart, Petria Thompson, Clifton David Fuller, Ethan Bernard Ludmir, Michael Kevin Rooney

**Affiliations:** 1 Department of Radiation Oncology The University of Texas MD Anderson Cancer Center Houston, TX United States; 2 Department of Radiation Oncology Johns Hopkins University Baltimore, MD United States; 3 Department of Radiation Oncology Dartmouth University Lebanon, NH United States; 4 Department of Radiation Oncology Texas Tech University Lubbock, TX United States; 5 Department of Radiation Oncology University of California San Francisco San Francisco, CA United States

**Keywords:** academic publishing, article processing charge, open access, oncology, open access publishing, scholarly communication

## Abstract

**Background:**

Open access (OA) publishing represents an exciting opportunity to facilitate the dissemination of scientific information to global audiences. However, OA publishing is often associated with significant article processing charges (APCs) for authors, which may thus serve as a barrier to publication.

**Objective:**

In this observational cohort study, we aimed to characterize the landscape of OA publishing in oncology and, further, identify characteristics of oncology journals that are predictive of APCs.

**Methods:**

We identified oncology journals using the SCImago Journal & Country Rank database. All journals with an OA publication option and APC data openly available were included. We searched journal websites and tabulated journal characteristics, including APC amount (in US dollars), OA model (hybrid vs full), 2-year impact factor (IF), H-index, number of citable documents, modality/treatment specific (if applicable), and continent of origin. All APCs were converted to US-dollar equivalents for final analyses. Selecting variables with significant associations in the univariable analysis, we generated a multiple regression model to identify journal characteristics independently associated with OA APC amount. An audit of a random 10% sample of the data was independently performed by 2 authors to ensure data accuracy, precision, and reproducibility.

**Results:**

Of 367 oncology journals screened, 251 met the final inclusion criteria. The median APC was US $2957 (IQR 1958-3450). The majority of journals (n=156, 62%) adopted the hybrid OA publication model and were based in Europe (n=119, 47%) or North America (n=87, 35%). The median (IQR) APC for all journals was US $2957 (1958-3540). Twenty-five (10%) journals had APCs greater than US $4000. There were 10 (4%) journals that offered OA publication with no publication charge. Univariable testing showed that journals with a greater number of citable documents (*P*<.001), higher 2-year IF (*P*<.001), higher H-index (*P*<.001), and those using the hybrid OA model (*P*<.001), or originating in Europe or North America (*P*<.001) tended to have higher APCs. In our multivariable model, the number of citable documents (β=US $367, SD US $133; *P*=.006), 2-year IF (US $1144, SD US $177; *P*<.001), hybrid OA publishing model (US $991, SD US $189; *P*<.001), and North American origin (US $838, SD US $186; *P*<.001) persisted as significant predictors of processing charges.

**Conclusions:**

OA publication costs are greater in oncology journals that publish more citable articles, use the hybrid OA model, have a higher IF, and are based in North America or Europe. These findings may inform targeted action to help the oncology community fully appreciate the benefits of open science.

## Introduction

Open access (OA) publication grants any individual permission to view published scientific articles without cost, thereby allowing free public access to content regardless of geography or consumer affiliation [1]. This contrasts with traditional subscription-based publishing which either requires institutional subscriptions to grant journal access or requires fees for access to individual articles. Various OA publication models exist, including Libre OA, Gratis OA, Gold OA, Green OA, Hybrid OA, Delayed OA, Academic Social Networks, and Black OA, among others. Each model has subtle differences in timing, cost, use restriction, and availability of published articles [2]. However, for practical purposes and the goal of this study, academic journals, in general, can be considered within 2 broad categories: “hybrid OA” refers to subscription-based journals that allow authors an option of making individual articles OA, whereas “full OA” journals publish exclusively OA content [3].

OA publishing allows for advancements in science and medicine to be adopted at faster rates and promotes the democratization of knowledge [4]. As such, there has been a rapid growth in OA publishing, with an estimated average growth of 30% in the number of published articles per year since the early 1990s [5]. Clinicians and patients with complete access to relevant studies can make more informed health decisions, rather than attempting to base their choices solely on the subset of studies from which they have access. Indeed, recent evidence has shown that readership increases significantly when an OA platform is adopted, an issue exemplified during the recent COVID-19 pandemic when the public was in need of high-quality medical literature [6]. Given the practical and ethical advantages of OA publishing, many research funding groups, including the National Institutes of Health, have mandated OA publication of funded research [7]. Further, in response to recommendations from research communities such as the Efficiency and Standards for Article Charges Initiative, many institutions and journal publishers have recently signed OA Transformative Agreements, which mandate an increased transition to OA article publication over a specified period of time [8].

Despite the clear advantages of OA publishing, there are also some potential disadvantages. Many journals require authors to pay article processing charges (APCs) in order to publish an OA manuscript. These fees can be significant, in the ranges of thousands of US dollars and thus may inadvertently serve as a barrier to OA publication, particularly for authors with limited personal or institutional resources [9,10]. To address this concern, some publishers have introduced APC waivers to authors from low- and middle-income countries. However, the adoption of these policies has been mixed, with premier widely read journals often being less likely to offer waivers [11].

In this study, we sought to identify and characterize journal-level factors associated with APCs in oncology journals. We hypothesized that journals with higher impact factors (IFs), those based in the United States, and those adopting the hybrid OA model would have higher APCs. Our results could be used to inform policy-level change aimed at promoting equity and equality in access to oncologic research.

## Methods

### Journal Identification

The SCImago Journal and Country Rank database [12] was queried on August 19, 2020, to identify oncologic journals. The following search parameters were used:

- Subject area: “Medicine”
- Category: “Oncology”
- Region or country: “All regions/countries”
- Types: “Journals”
- Year: “2019”

The resulting journals were screened according to OA publishing status. Journals with an OA publishing option (hybrid or full) and APC data available via their website were included. Journals that were discontinued or written in non-English language were excluded from this analysis. Journals with less than 10 published articles per year were also excluded.

### Journal Evaluation

For each journal meeting inclusion criteria, the journal’s website was manually searched, and the following data points were tabulated: APC (in US $), OA model (hybrid or full), 2-year IF, H-index, number of citable documents, modality specificity (yes or no), treatment site specificity (yes or no), and continent of origin (North America, Europe, or other). A journal was considered modality specific if its stated mission and scope were to publish content related to a particular oncologic treatment modality (eg, surgery, radiation therapy, etc). Similarly, journals were considered site specific if the stated mission and scope were related to a particular disease site (eg, breast cancer, colorectal cancer, etc), as opposed to oncology in general. All APCs were converted to US-dollar equivalents for final analyses. An audit of a random 10% sample of the data was independently performed by 2 authors (MKR and UGG) to ensure data accuracy, precision, and reproducibility.

### Statistical Analysis

Data were summarized using descriptive statistics. Pearson correlation was used to identify associations between continuous variables. Wilcoxon rank sum and Kruskal-Wallis testing were used to identify differences in continuous variables. For factors with significant associations on univariable testing, a multiple regression model was created to identify factors independently associated with APCs. For collinear factors, only 1 was used in the multivariable model. Log transformation was used for right-skewed predictive variables. All analyses were performed with R 4.0.3 (R Foundation for Statistical Computing). Data collection and analysis were performed from August 19, 2020, to December 11, 2021.

### Ethical Considerations

Institutional review board approval was not necessary for this study and was waived accordingly. Informed consent was not required as the included information is publicly available.

## Results

Of 367 oncology journals identified in the SCImago database, 251 met the final inclusion. The most common reasons for exclusion were journals not having an OA publishing option or not having detailed information regarding APCs on the journal website. The majority of journals (n=156, 62%) adopted the hybrid OA publication model and were based in Europe (n=119, 47%) or North America (n=87, 35%). The median APC for all journals was US $2957 (IQR 1958-3540). Twenty-five (10%) journals had APCs greater than US $4000. There were 10 (4%) journals that offered OA publication with no publication charge. The median number of citable documents per year was 308; however, there was significant variation in publication volume across evaluated journals. About 14% (n=35) of journals published fewer than 100 articles per year, while 11% (n=27) published at least 1000 citable articles per year. North American (median 3.1, IQR 2.3-5.0) and European (median 3.0, IQR 2.1-4.6) journals tended to have a greater IF than journals from other regions (median 2.3, IQR 1.5-2.9; *P*<.001). There were no significant differences in IF between full OA (median 2.8, IQR 1.7-3.9) and hybrid OA journals (median 2.8, IQR 2.2-4.2; *P*=.28).

Table 1 summarizes journal baseline characteristics and the results of the univariable and multivariable models to identify predictors of publication costs. Increased number of citable documents (*P*=.006), higher IF (*P*<.001), use of the hybrid OA model (*P*<.001), and North American origin (*P*<.001) were independently associated with increased APCs according to the multivariable model. Beta coefficients are provided in Table 1 to estimate the effect of various independent variables on APCs. For every 10-fold increase in the number of citable documents and journal IF, there is an estimated increase of US $367 and US $1144 in APC, respectively. The use of the hybrid OA model was associated with an increase of US $991 in APCs. Furthermore, compared to European journals, North American journal APCs were predicted to be US $838 higher.

**Table 1:** Univariable and multivariable models to identify factors associated with journal article processing charges.

Characteristic		Univariable model		Multivariable model	
		Median (IQR) or n (%)	*P* value	Beta (SE) (US $)	*P* value
Article processing charge (US $)		2957 (1958-3540)^a^	N/A^b^	N/A	N/A
Mean number of citable documents per year^c^		308 (140-536)^a^	<.001	367 (133)	.006
Two-year impact factor^c^		2.8 (2.0-4.1)^a^	<.001	1144 (177)	<.001
H-index^d^		49 (30-89)^a^	<.001	N/A	N/A
**Site specific**					
	Yes	77 (31)^e^	.94	N/A	N/A
	No	174 (69)^e^	N/A	N/A	N/A
**Modality specific**					
	Yes	35 (14)^e^	.45	N/A	N/A
	No	216 (86)^e^	N/A	N/A	N/A
**Journal type**					
	Full OA^f^	95 (38)^e^	<.001	N/A	N/A
	Hybrid OA	156 (62)^e^	N/A	991 (189)	<.001
**Region**					
	Europe	119 (47)^e^	<.001	N/A	N/A
	North America	87 (35)^e^	N/A	838 (186)	<.001
	Other	45 (12)^e^	N/A	–307 (213)	.15

^a^Median (IQR).

^b^N/A: not applicable.

^c^Due to the presence of outliers, these variables were log-transformed for multivariable linear modeling.

^d^Due to collinearity with the impact factor, the H-index was not included in the multivariable model.

^e^n (%).

^f^OA: open access.

**Figure 1:**
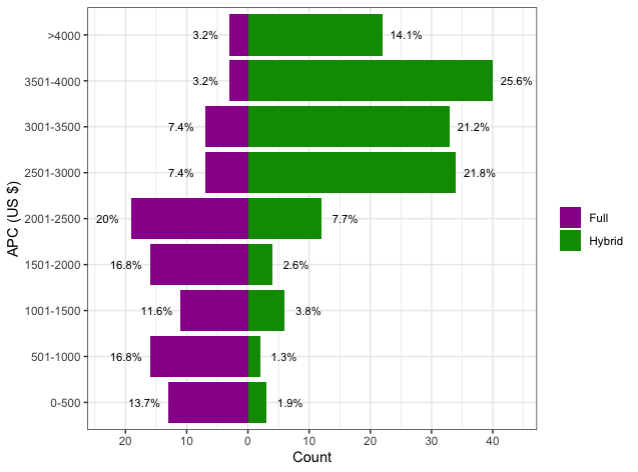
Distribution of article processing charges (APCs) displayed separately for journals adopting full and hybrid open access publishing models.

Figure 1 describes the distributions of APCs for all identified journals, displayed separately for hybrid OA and full OA journals. Overall, hybrid journals had higher publication costs than their full OA counterparts. The median APCs for hybrid and full OA journals were US $3260 and US $1958, respectively. Only 11% (n=17) of hybrid journals charged less than US $2000 for OA publication, compared to 59% (n=56) of full OA journals. Furthermore, 14% (n=22) of hybrid journals had APCs of at least US $4000, while only 3% (n=3) of full OA journals charged this amount.

**Figure 2:**
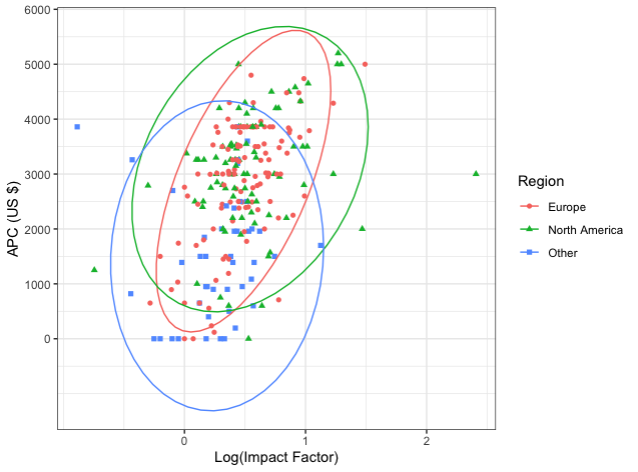
Association between impact factor and article processing charge (APC) among journals housed in various global regions.

The associations between APC, journal IF, and region of origin are summarized in Figure 2. A higher journal IF was associated with increased APCs; in general, journals based in Europe and North America were more likely to have a higher IF and publication costs. Only 21% (n=25) and 18% (n=16) of European and North American journals had an IF less than 2, respectively, compared with 42% (n=19) of journals housed in other regions.

## Discussion

### Principal Findings

In this cross-sectional analysis of OA publication practice among oncology journals, we not only found that a significant portion of journals charged high fees for publication but also identified multiple journal-level factors associated with higher costs. In particular, our data suggest that journals using the hybrid OA model, having a higher IF, publishing a higher volume of articles, and those housed in North America tend to charge more for authors to publish their articles openly. Taken together, these findings suggest that current publication standards in the field of oncology may limit sharing of knowledge, particularly among select journals. These data may ideally be used to inform policy-level changes aimed at improving equitable dissemination of knowledge among the oncologic community.

The OA publication model is intended to increase the dissemination of scientific findings by bypassing consumer financial barriers to article access. Indeed, prior research has shown that articles published in an OA forum are more likely to be viewed and downloaded compared to papers limited by a paywall [13,14]. Furthermore, growing evidence has shown that OA articles are more likely to be cited in the subsequent peer-reviewed literature, a phenomenon termed the “open access citation advantage,” suggesting increased engagement within the scientific community [15-17]. However, the benefit of open publishing is not limited to the scientific or medical community alone. Particularly within the medical field and oncology, OA publishing creates an opportunity for improving patient education, advocacy, and shared decision-making. Access to peer-reviewed literature may deter patients from seeking information from less reliable sources and may further improve public trust in science and medicine. Finally, because medical research is often supported through public funds, the public may have a strong interest in accessing science in their role as funders, advocates, and research participants [18].

Plan S is an OA initiative that stipulates research derived from public grant funding must be published in a full and immediate open manner. This enterprise is supported by cOAlition S, a consortium of international research funders and research-performing organizations. The objective of Plan S is to not only increase the number of articles published in OA journals but to ensure public funding directly leads to research that the public can easily access. For full compliance with Plan S, authors must publish in OA journals or platforms, publish in subscription journals and choose to make the articles available OA (but cOAlition S will not financially support OA charges) or publish OA in subscription journals under a transformative agreement (subscription publishers agree to transition to OA by a given date). In addition, Plan S is working on a journal comparison service, which would increase transparency in OA journals by allowing APCs to be directly compared.

While cOAlition S is already supported by the European Commission and the European Research Council, the United States has not yet adopted these standards. The United States continues to rely heavily on traditional publishing methods that require reader subscriptions to access articles. Encouragingly, a recent policy from the US government has required the implementation of OA publication starting in 2026, which represents a promising change in value prioritization [19].

It is worthwhile to note that Plan U (Plan Universal), a research preprint initiative, offers a unique compromise in the realm of OA versus traditional publishing methods. Plan U advocates for all funding agencies to mandate research preprints, which would essentially eliminate the time between submission and official publication. Preprints would be instantaneously uploaded on the web, which would not only allow for an expedited peer-review process from a larger number of individuals but would give everyone access to new and potentially impactful research [11]. Researchers would still be able to publish in subscription-based journals but would additionally release the manuscripts on preprint websites.

Open publication is still the optimal avenue for the sharing of many, if not all, scientific articles. However, many barriers exist that may limit the ability of researchers and authors to publish their work under current publishing models. Most notable is the presence of publication costs in the form of APCs, which authors often must pay themselves in order to publish their work, provided their employer or affiliated institution has not signed a transformative agreement to cover costs of publication while transitioning to full OA. This can be particularly burdensome for individuals with limited personal or institutional resources, as costs of publication can be quite high. In the present study, we found that the median APC was nearly US $3000, with 10% of oncology journals charging more than US $4000 for the OA publication of a single article. Many institutions and academic groups have developed strategies to address APCs, such as pooled funds dedicated specifically to submission fees. Furthermore, some journals have instituted waiver policies for financially vulnerable populations, including submitting authors from low- and middle-income countries. However, the adoption of these policies has not been universal, and there is variation in the extent of discounts across journals [11].

Significant publication costs not only create the problem of limiting shareability of the science itself, but they also create a structural framework that predisposes to academic inequity. Authors that are fortunate to have available personal funds or strong institutional support may elect to pursue OA publication and therefore reap the benefits described previously, including improved visibility. In contrast, authors unable to afford OA publication fees may need to publish their work behind a paywall or, worse, may have limited options to share their findings in a scientific journal altogether. Consequently, this may create a cyclical disparity in academic opportunities wherein disadvantaged authors are further limited in their ability to participate in and share scientific research.

The optimal solution to the financial crisis of OA publishing likely will not involve consumer strategies to obtain funds to meet APC requirements but rather focus on reform of publication practice at large, as evidenced by recent efforts on behalf of governments and professional scientific groups to mandate a transition to OA publication in the coming years. Our study provides initial support that certain journal characteristics may be associated with an increased risk of high publication costs; these findings may encourage further investigation into potential strategies that might encourage a more equitable publishing economy.

One of the most important trends identified in this analysis is the association between journal IF and APCs (Figure 2). We found that for every 10-fold increase in IF, there was an estimated increase of US $1144 in this population (Table 1). Although IF is an imperfect metric, higher-impact journals are often desired forums for publication of scientific work, as they may offer increased article visibility and credibility [20]. Journals with a higher IF typically receive a higher volume of submissions and may be more selective in editorial acceptance decisions. Such journals, therefore, also may be afforded the ability to charge higher APCs as authors may be more willing to pay more to have their articles freely accessible in a highly visible and respected environment. Furthermore, it is possible that high-impact journals may more often publish work from research groups with greater private or institutional resources and, thus, OA APCs are less limiting among this population of researchers. Regardless of the underlying etiology explaining this trend, the strong association between IF and APCs warrants further exploration with a reevaluation of APC policies, particularly among the highest-impact oncology journals.

Our analysis also revealed a strong relationship between the type of OA publication model and processing costs (Figure 1). At the time of data collection, the majority (62%) of journals used a hybrid model wherein authors are offered the option of publishing OA after paying an APC. We found that hybrid journals tended to have significantly higher OA publication costs, with an estimated difference of US $991 compared to their full OA counterparts (Table 1). Importantly, unlike journal IF, region of origin, and number of published documents, hybrid OA status does not have an obvious relation to publisher costs, and thus represents a promising opportunity for immediate improvement. The trend likely reflects the impact of choice on author submission preferences. For full OA journals publishing exclusively OA papers, authors are not granted any choice and thus every submitting author is required to pay a publication fee. In contrast, with the hybrid model only authors with significant interest in having their article benefit from open publication and, more importantly, those willing and able to pay the APC will elect for OA publication. As a result, journals adopting the hybrid model may be able to be more stringent in their APCs without deterring potential submissions. As described above, however, this pattern likely does introduce biases that hinder the ability of authors with limited resources to participate in open science. Therefore, we argue that even though authors are granted a choice of participation in the hybrid model, these journals may actually benefit the most from modification of publication standards to be less financially exclusionary of authors from differing backgrounds. Further, we propose that authors thoughtfully consider the implications of the hybrid publication model when choosing journals as potential forums for publication of their research.

While OA publishing models certainly do benefit consumers of science at large, there are important economic considerations regarding the adoption of open science that need to be recognized in order to fully contextualize the findings of this investigation. Compared to traditional subscription-based publishing costs, which are often covered by a library or institutional contracts, APCs for OA publication benefit from increased cost transparency for consumers of scientific articles. Regardless of the publishing model, however, there will inevitably be costs associated with the evaluation, review, and publication of scientific findings, such as hiring of editorial and production staff in addition to the peer-review process. Although these costs are not universal across journals (eg, some editorial positions are purely voluntary without compensation), any expenses will inevitably need to be met in order for science to be vetted and published responsibly. APCs are 1 practical method of meeting these costs and, therefore, do represent a feasible mechanism to allow for free public access to scientific work. However, APCs themselves are likely derived, at least in part, from costs specific to any given journal. For example, editorial salaries would be expected to be higher in journals that have a greater number of submissions or in areas with a greater cost of living such as North America and Europe. It is, therefore, critical that such factors are considered when interpreting the results of this study. Despite any unadjusted confounding factors related to APCs and journal characters, this investigation does provide hypothesis-generating data which may be used to stimulate further research in this area with the goal of improving equitable open science.

This investigation is limited by several factors related to the study design. First, as with any observational study, evaluation of causal relationships between variables is difficult. However, we did attempt to consider all publicly available data with intuitive potential impact on APCs when generating our multivariable model. Second, some endpoints, such as the proportion of papers published OA in hybrid journals, were of interest for the scope of this investigation but were not readily available on the web and thus may limit our ability to analyze patterns in author submission preferences. Last, although we did survey all oncology journals listed in the SCImago database, a minority of journals were excluded either due to lack of transparent APC information or non-English language, which may limit the generalizability of our findings.

### Conclusion

OA publication has been shown to improve article visibility compared to traditional subscription-based publication; however, APCs can pose a significant burden to authors and researchers interested in publishing their work in an OA forum. In this cross-section observational study of publication practices among oncology journals, we find that APCs were greater in journals with a higher IF, more citable documents, those originating in North America, and those utilizing the hybrid OA model. These results warrant further investigation and reevaluation of publication standards which ideally may promote equitable sharing of oncologic research and knowledge.

## Abbreviations

APC: article processing charge
IF: impact factor
OA: open access

## Data Availability

The data sets generated during and/or analyzed during the current study are available from the corresponding author on reasonable request.
